# The growing oversupply of physicians in Ecuador: challenges and implications for the healthcare system

**DOI:** 10.3389/fpubh.2025.1605845

**Published:** 2025-08-18

**Authors:** Jorge Vasconez-Gonzalez, Juan S. Izquierdo-Condoy, Luis Merlo, Bernardo Sandoval, Esteban Ortiz-Prado

**Affiliations:** ^1^One Health Research Group, Faculty of Health Science, Universidad de Las Americas, Quito, Ecuador; ^2^Dean of the Faculty of Medicine at Universidad de Las Américas, Quito, Ecuador; ^3^Medical Director of the Metropolitan Specialties Hospital, Quito, Ecuador

**Keywords:** oversupply, physicians, public health, healthcare system, Ecuador, health workforce, physician distribution, geographic disparity

## 1 Introduction

As part of the United Nations' Sustainable Development Goals, Goal 3 prompts subscribed countries around the globe to ensure healthy lives and promote wellbeing for all throughout all ages (1). In order to achieve this, countries must strive to develop a strong and functioning health system that can not only cover the demands of their population, but that can work efficiently to ensure their own sustainability. In this context, policies surrounding the training, development and geographical distribution of a country's health workforce represent an important axis in their ability to achieve this goal. Critical among these is the proportion of healthcare workers for a given population, which varies depending on region, demographic composition, and median income.

Although the World Health Organization (WHO) widely promotes the benchmark of at least 23 health workers per 10,000 populations as the minimum threshold necessary to deliver essential health services (2–4), this figure does not specifically refer to physicians. In fact, the recommendation encompasses a broader category that includes doctors, nurses, and midwives. Despite this, the figure is often interpreted as a target for physician density, although no globally accepted reference exists for the ideal number of physicians per population. According to World Bank data, countries vary significantly in how they meet this threshold depending on their income level. High-income countries report ~3.3 physicians per 1,000 populations, while upper- and lower-middle-income countries report 2.2 and 0.7, respectively (2). This excess or deficit of healthcare professionals can be one of the main problems faced by health systems since estimating the real needs of the system (and by consequence, the proportion of physicians needed for each region) is a complicated process for which there is no single accepted method (4, 5).

In Ecuador, data from the National Institute of Statistics and Censuses (INEC) for 2023 indicate that non-communicable diseases were the leading causes of mortality, with ischemic heart diseases (*n* = 13,318), cerebrovascular diseases (*n* = 4,632), and diabetes mellitus (*n* = 4,460) topping the list. Among infectious diseases, influenza, and pneumonia (*n* = 3,781) were major contributors to mortality. Other significant causes of death included assaults (*n* = 7,308) and traffic accidents (*n* = 3,965) (6). The Ecuadorian healthcare system manages these health burdens through its public and private sectors. The public sector comprises the Ministry of Public Health, the Ministry of Economic and Social Inclusion, municipal health services, and social security institutions such as the Ecuadorian Social Security Institute (IESS), the Armed Forces Social Security Institute, and the National Police Social Security Institute. The private sector includes both for-profit and non-profit organizations (7).

Nationwide, there are 4,148 healthcare establishments, of which 631 are inpatient (hospital) facilities and 3,517 are outpatient centers (8). Approximately 80% of these facilities belong to the public sector: the Ministry of Public Health operates 47%, and the IESS manages 24%. The remaining public facilities are operated by municipalities, other ministries, and various social security institutions. The private sector accounts for roughly 20% of healthcare establishments, divided between 14% for-profit institutions and 6% non-profit organizations (7).

Regarding health coverage, only 32.9% of the Ecuadorian population has access to some form of health insurance, with coverage rates of 30.7% among women and 35.2% among men. Among the insured, 4,279,738 individuals are covered by the IESS general insurance, 69,971 hold private insurance with a policy, and 10,958 have private insurance without a formal policy. Nevertheless, ~12,030,720 Ecuadorians remain uninsured (9).

**Figure 1:**
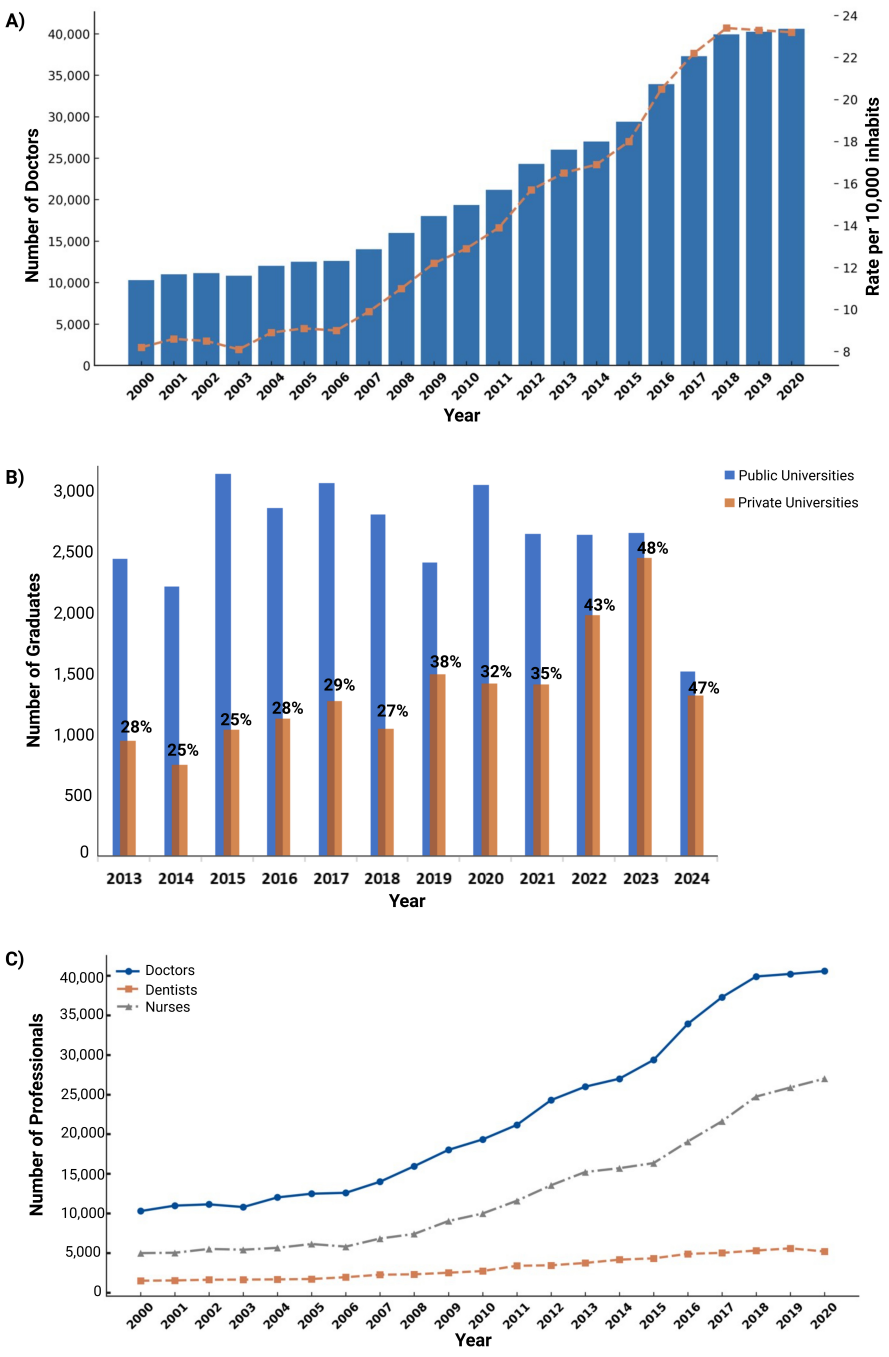
Trends in the Growth and Composition of the Healthcare Workforce in Ecuador (2000–2024). **(A)** Trends in the Number of Doctors and Rate per 10,000 Inhabitants in Ecuador (2000–2020). The bar chart (Lancet blue) represents the total number of doctors in Ecuador each year, while the line graph (dark peach) indicates the rate of doctors per 10,000 inhabitants. The data show a steady increase in both the number of doctors and the rate over the two decades, reflecting improvements in healthcare workforce availability. Data extracted from the last update of the INEC (42). **(B)** Annual Number of Graduates from Public and Private Universities in Ecuador (2013–2024). The blue bars represent the number of graduates from public universities, while the dark peach bars indicate graduates from private universities. The percentage labels above each bar represent the proportion of graduates from private universities relative to the total for that year. Over the years, the proportion of private university graduates has increased, reaching 48% in 2023 and 47% in 2024. Data was extracted from the last update of SENESCYT (14). **(C)** Growth of Healthcare Professionals in Ecuador (2000–2020). This stacked bar chart represents the total number of healthcare professionals over time, categorized by doctors (Blue), nurses (Neutral Gray), and dentists (Dark Peach). The visualization highlights the increasing workforce in Ecuador's healthcare system, showing a notable rise in the number of professionals across all three categories. Data extracted from the last update of INEC (42).

Physician density in Ecuador has shown notable growth over recent decades. Between 2000 and 2017, the physician-to-population ratio increased from 0.8 to 2.2 per 1,000 inhabitants (4). In 2016, the national physician proportion per 10,000 inhabitants was 20.52 and in 2017 it climbed up to 22.2 (Figure 1A). In his work, Hidrobo mentions that currently, the physician rates stand at around 38.47 per 10,000 inhabitants. If this trend would continue, it would lead to an important risk of physician overpopulation by 2030, as the national medical population grows from 33,925 physicians (or 20.5 per 10,000 inhabitants) in 2016 to 140,734 by the year 2030 (59.1 per 10,000 inhabitants) (10, 11).

According to data from the Secretariat of Higher Education, Science, Technology, and Innovation (SENESCYT), the largest volume of new physicians that have graduated from 2013 to 2024 have come from public universities. However, the data also shows a clear trend toward the closing of this gap, with a slow decrease in the relative number of graduates from public universities and an increase of those hailing from private institutions (Figure 1B).

When comparing the number of medical doctors since 2000 with other health professionals such as nurses or dentists, we can see that the number of physicians has increased the most over the last two decades (Figure 1C). This trend coincides with periods of time in which the country increased its number of medical schools in a disproportionate manner to its population growth. Before the year 2000, Ecuador had nine functioning medical schools, most of which were located in large capital cities. In the following 10 years, this number increased by 10 and then by eight more by 2023. As of 2023, state reports show 28 functioning medical schools registered throughout the country, some of which have reported student populations up to 6,379 students in the last 10 years (12–14). This increase in the number of schools is believed to be the result of an increased interest in medical education and rising entry requirements for public schools' admissions programs. This generalized interest is reflected in the number of applications for medical schools throughout the country. For example, data from 2022 showed 22,449 registered applications for that year alone (13, 15).

**Figure 2:**
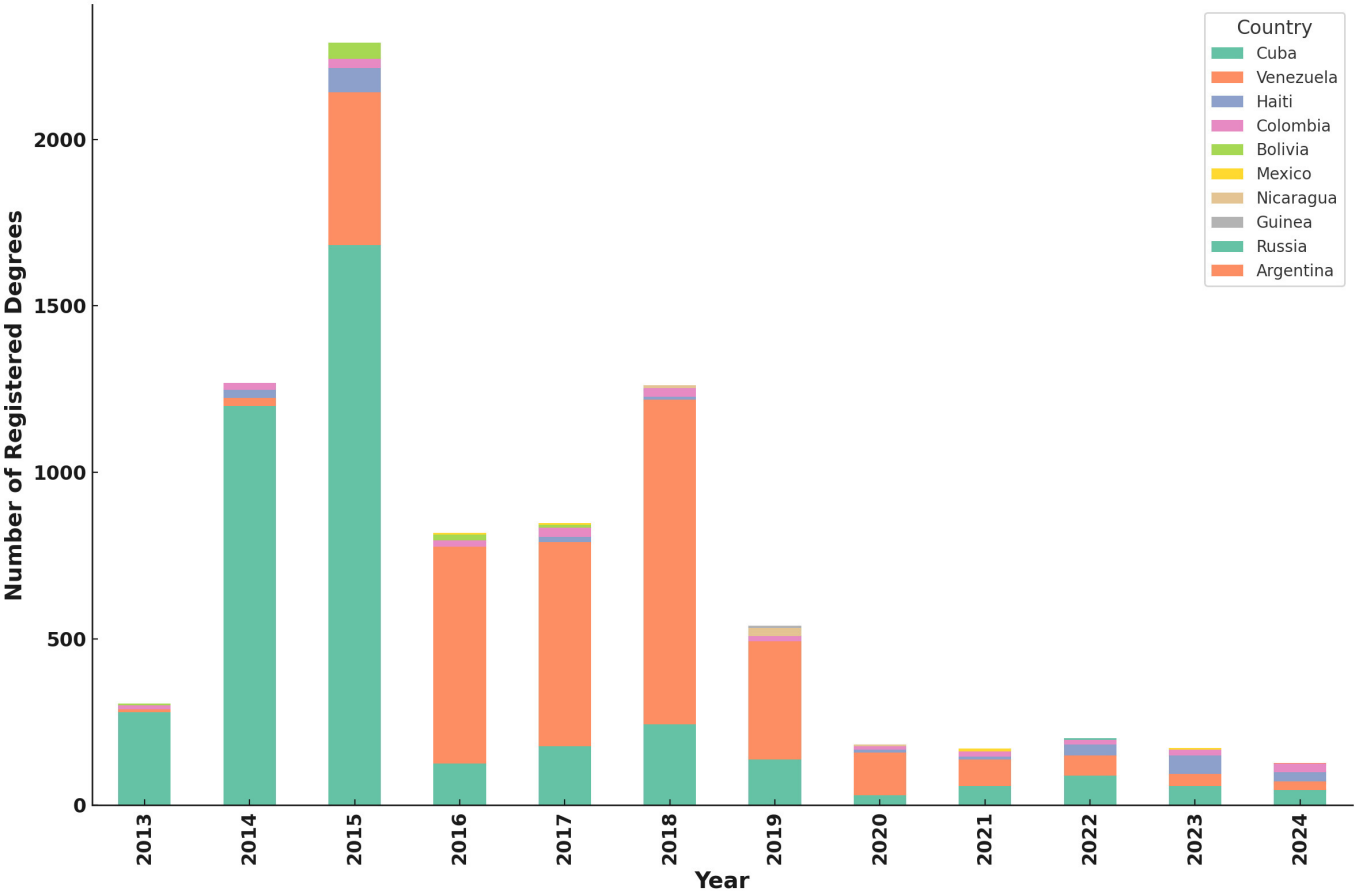
General medical degrees from abroad registered in Ecuador from 2013–2024.

In parallel, the inflow of foreign-trained physicians and foreign nationals seeking to practice in Ecuador must also be considered. According to data from the SENESCYT, between 2013 and 2024, a total of 8,555 foreign academic degrees were registered. Of these, 1,952 (22.8%) corresponded to Ecuadorian citizens who pursued studies abroad and subsequently validated their degrees nationally, while 6,603 (77.2%) were registered by foreign nationals. Early in this period (2013–2015), Ecuadorian registrations predominated, but beginning in 2015, foreign registrations surged, largely driven by migration waves from Cuba and Venezuela. For instance, by 2015, foreign nationals represented over 91% of all degree registrations. Cuban nationals predominated early registrations (2014–2015), whereas Venezuelans became the majority from 2016 onwards, mirroring regional political and economic crises. In more recent years, although overall registration numbers have declined, the diversity of countries represented has broadened, with significant contributions from Haiti, Nicaragua, Mexico, and Russia. Notably, most registered degrees were postgraduate qualifications (44 fourth-level degrees), compared to only one technical degree, indicating a trend toward higher academic specialization among both returning Ecuadorians and foreign professionals (14) (Figure 2).

Regarding the emigration of Ecuadorian healthcare workers, official national statistics remain unavailable. However, a study by Ayora et al. (16) reported that, as of 2007, Ecuadorian health professionals constituted 59.1% of all foreign-trained medical personnel in Chile.

Naturally, the increase in the number of faculties does not necessarily correlate with an increase in the quality of education. In Ecuador, medical licensing examinations formally began in 2014, under the Higher Education Quality Assurance Council (then CEAACES, now CACES) providing, for the first time, a benchmark by which to assess end-career results for each medical school. National results from the last decade show a relatively high percentage of physicians who fail to achieve a passing grade in this exam, despite having graduated successfully from their respective Universities (17). To date, an estimated 50 to 70% of physicians who take this exam for the first time are successfully licensed, but these results vary greatly from institution to institution and between regions. Sadly, this has resulted in ~20%−30% of graduated physicians not being able to obtain their license, with some cases reportedly failing to do so up to 16 times (18, 19). This fact is worsened by the influx of foreign physicians that have immigrated as part of government programs and as the result of humanitarian crises in the region. These professionals often enter a saturated job market and most fail to pass their licensing examination, causing the unlicensed physician problem to bloat (20). For instance, medical licensing results for physicians attempting to legalize their foreign medical degrees had the lowest approval rate for the 2024 examinations, achieving only a 29% approval rate (21). This would imply that the problem may not wholly lie on medical education in the country, but on how the medical system is operating with its related sectors.

It is important to acknowledge that licensing examination scores are not absolute indicators of clinical competence. Although evidence suggests that scores like USMLE Step 2 CK moderately predict residency performance, multiple demographic, psychosocial, and administrative factors can influence outcomes (22, 23). Nevertheless, Ecuador lacks a comprehensive national strategy to assess and guarantee physician competency, resulting in notable heterogeneity across graduates.

Beyond concerns about patient care quality, physician oversupply has led to deteriorating working conditions for both newly graduated and practicing physicians. The mismatch between the number of new medical graduates and the availability of job opportunities forces many to accept precarious employment, adversely impacting healthcare delivery (24–26). Data from the Ecuadorian College of Physicians indicate that ~1,500 doctors graduate each year, yet only 100 to 150 new job positions are created annually (12). In Guayas province, home to an estimated 12,000 medical professionals, only about 50% report holding stable employment. This challenge is not unique to Ecuador; other countries in the region similarly struggle to establish appropriate quotas for training new healthcare professionals (27).

This trend predominantly affects general practitioners, who complete a 6-year medical baccalaureate program, while the opposite is observed in most medical specialties (25). However, existing residency training programs are insufficient to accommodate the growing demand, leaving many recent graduates to spend years awaiting entry into specialty training or, alternatively, seeking opportunities abroad (10). This situation is exacerbated by limited funding for residency scholarships and an inadequate number of training positions proportional to national healthcare needs. Additionally, these constraints have extended to undergraduate education, as budgetary and infrastructure limitations increasingly hinder access to rotating internship placements during the final year of medical school (11).

## 2 Geographic distribution of medical doctors

In Ecuador, as in most other countries, geographic distribution of professionals makes the planning of healthcare workforce supply a complicated manner. There is an international trend of physicians migrating toward urban zones in search of better economic conditions and accommodation. This has motivated governments to implement incentives programs to supply healthcare workers to rural populations. However, evidence from rural programs in the United States suggests that low-income counties outside of the first poverty quartile receive less attention from physicians who seek for better benefits coming from working in more impoverished areas (28), leaving low to middle-income counties undersupplied. In Ecuador, one such program is the community medicine program, colloquially known as “rural medicine.” Sadly, while mandatory, this only includes recent graduates who, as discussed, are inexperienced and ever more often prefer to skip the program overall for safety concerns, adding to the population of physicians without a legal license to practice (29). Trends like these negatively affect rural population health and life expectancy as proper, specialized healthcare becomes harder to access (30).

The distribution problem of the Ecuadorian healthcare workforce is highlighted when analyzing the data over the years. By analyzing physician supply by province, it becomes evident that rates per 10,000 inhabitants have increased globally. However, this increase is not homogeneous and may not represent the necessities of a particular population. The provinces with the highest increase in physician supply were Pastaza, Zamora-Chinchipe, and Galápagos, which went from rates of 11.3, 9.6, and 7.1 in 2006 to 40.4, 33.2, and 31.0 in 2020, respectively. This increase is not, however, due to a high brute physician output, but to the province's demographic characteristics and local population not increasing in par with the influx of new medical personnel. In provinces with larger populations, such as Pichincha, Guayas, and Manabí, rates have increased more slowly, with rates of 26.9, 22.9, and 23.5, respectively (Supplementary Figure S1). Inversely, rural provinces like Santa Elena, Esmeraldas, and Los Ríos still suffer from lack of medical personnel for their populations' needs. This makes it clear that coverage is not met despite the increase in medical trainees, and that policies from the last two decades have led to a polarization of professionals, with surplus in urban areas and understaffing in rural ones.

To address this issue, emerging technologies such as artificial intelligence (AI) offer promising solutions. Telemedicine platforms equipped with AI-powered chatbots and virtual assistants can facilitate remote consultations, provide health information, and assist in the interpretation of diagnostic imaging such as X-rays and CT scans. By enhancing diagnostic accuracy and supporting early disease detection, these tools have the potential to significantly expand access to healthcare services in underserved regions (31).

## 3 A call to action

There is no clear solution to this problem, since estimations of healthcare worker's needs are not definitive or agreed on. As has been seen, increasing the supply of physicians can cause a surplus in already packed populations, with little impact on those with less income. However, limiting quotas for new students poses a risk of worsening the situation for understaffed rural centers. This is particularly important in a country like Ecuador, where most health personnel working in rural areas are the product of the government's community medicine program. There is also the problem of establishing the proper quota for any health system. For instance, necessities in OECD countries vary greatly and predictions for 2030 show an important surplus from countries like Mexico and Germany, but important shortages in the United States and France (32). However, even these estimates have a hard time when accommodating migration and sudden public health crises like the COVID-19 pandemic. Finally, algorhythms should be careful to account for public spending and working days for physicians, since these factors can significantly alter long-term goals for a health system (33).

Naturally, there is also the “quality of education problem,” since mass education poses a risk of graduating underperforming professionals. A historical view on policies that have led to the closure of underfunded medical schools or unified curricula shows they have been successful in homogenizing medical graduates but have also led to further centralization and diminishing support for vulnerable populations and minorities. This is clear from the effects of Dr. Abraham Flexner's report on medical education in the United States and Canada, published in 1910, and at the time one of the major drivers of the country's pursuit of a highly controlled and centralized medical education system (34). While the positive effects of this were clear in the quality of new medical graduates in the new American system, negative effects included a bias toward a biomedical model, loss of focus on medical humanities and the loss of opportunities for rural populations, minorities, and women to formally train in medicine (35, 36). This poses a significant challenge for health and education officials who must now seek a balance between laxity and severity in their policies.

In the face of this complex problem, the authors consider it necessary that public officials formulate policies to properly regulate the academic offer of both undergraduate and postgraduate medical programs in line with the needs established by the national health authority. Additionally, evaluation and accreditation policies should ensure periodic evaluation of the training process, education laboratories, simulation centers, and teaching staff of existing medical schools (11, 24). Quality assurance processes should be based on international standards, such as the World Federation of Medical Education (WFME) accreditation policy, for which Ecuador's CACES is not yet a subscriber. Identification of medical schools that fail to provide adequate education environments or lack proper accreditation should be considered for closure (37). Additionally, both the Ecuadorian higher education authorities and the Ministry of Health must collaborate with existing medical associations to rethink the professional profile of the general physician and determine proper funding policies for medical residency programs in much needed areas (13). Finally, graduation requirements must be reconsidered throughout the country, as well as periodic evaluations that allow institutions to properly measure the achievement of the program's educational goals (38).

A failure to plan based on the country's real healthcare needs risks perpetuating physician unemployment. Consequently, it is imperative for the Council for Higher Education and the Ministry of Public Health to take decisive action. Key measures include reviewing medical training processes, incentivizing the relocation of specialists to underserved provinces, and addressing employability challenges. Currently, low employability discourages physicians from practicing in rural areas, leading to an overconcentration in major cities (39, 40). Additionally, an alarming trend has emerged wherein some hospitals prefer to utilize the unpaid labor of medical residents, reducing reliance on hiring general practitioners to cut salary expenses (41).

Postgraduate training availability is another critical issue. The limited number of residency positions compels many physicians to seek training opportunities abroad. Those who remain in Ecuador often face untenable working conditions under the current regulation of Teaching and Assistance Units, approved by the Council for Higher Education (CES) and the Ministry of Public Health. Postgraduate students are required to work 256 h per month without remuneration, and hours missed due to force majeure events are not waived but must be compensated through additional service (40, 41). This situation is unsustainable and discourages retention of medical talent within the country.

## 4 Future directions and priorities

### 4.1 Formulate public policies to regulate the offer of undergraduate and postgraduate degrees in medicine

These policies are urgently necessary. Currently, the country lacks a clear standard to evaluate the pertinence of new medical degrees. With free rein on new medical schools, smaller, underfunded, and understaffed programs risk increasing the already bloated physician population whilst failing to guarantee proper training. Needs should be properly established through active collaboration and conversation between education and health authorities, academia, and regulatory bodies.

### 4.2 Evaluation of training processes, laboratories, simulation centers, and teaching staff of medical schools

Accreditation is necessary to guarantee proper academic training, which involves appropriate staff and infrastructure. Policies to ensure responsible budget and financial planning for universities should protect students from programs closing suddenly due to lack of funding. CACES should subscribe to the WFMA accreditation policy and ensure that nationally accredited medical schools guarantee competence at an international level. Faculties that fail to be properly accredited must be considered for conditioning or closure.

### 4.3 Rethink the country's physician profile

Study plans should reflect the actual needs of the Ecuadorian population, but the current physician's profile is outdated. Without a proper update, the licensing examination risks being obsolete or disconnected from societal needs. If possible, licensing should go beyond clinical knowledge but should include clinical skills and interpersonal competence.

### 4.4 Admission and graduation requirements should be revised

Access to medical schools in Ecuador is diverse. Public schools take the “Ser Bachiller” exam, which is a verbal, numerical, logical, and abstract reasoning test, while private schools develop their own admissions processes. While protecting institutional autonomy, admissions processes should be properly enforced and reviewed by national authority. Admissions quotas should be revised in collaboration with the Health Ministry and graduation requirements should be reviewed. In the last decade, the minimum number of training hours for Ecuadorian medical students has reduced steadily, leading to much variation in teaching quality and less student-teacher contact.

## 5 Conclusion

Ecuador faces a growing oversupply of physicians due to the rapid expansion of medical schools and uncontrolled graduation rates, which are not aligned with labor market needs. This surplus is exacerbated by disparities in geographic distribution, with urban areas experiencing saturation while rural regions remain underserved. Additionally, high failure rates in licensing exams indicate significant gaps in medical education quality. The mismatch between general practitioners and specialist training opportunities further limits career progression, leading to underemployment and physician migration. Without strategic workforce planning, accreditation reforms, and expanded residency programs, Ecuador risks compromising healthcare quality, increasing physician unemployment, and destabilizing its health system.

## 6 Limitations

This study has several limitations. Although it highlights and analyzes the heterogeneity in the outcomes of the Professional Qualification Examination which all health professionals must take upon graduation and the proliferation of medical schools with varying levels of quality, it does not include a detailed assessment of institutional educational quality or provide comparative performance data across universities. The absence of such information limits the ability to more accurately identify the structural factors underlying the observed disparities in medical competencies.

Furthermore, this study relies exclusively on quantitative and documentary sources and does not incorporate qualitative perspectives from key stakeholders, such as health authorities, medical associations, professional colleges, health service employers, or physicians themselves. The inclusion of interviews, surveys, or focus groups to capture these perspectives could have enriched the discussion by reflecting the lived experiences of those most affected by physician oversupply, particularly regarding labor dynamics, working conditions, and perceptions of systemic shortcomings.

Nevertheless, despite these limitations, this article offers a comprehensive and timely assessment of the evolution of Ecuador's medical workforce and provides policy-oriented recommendations grounded in the available national data. Future research should complement these findings with qualitative approaches and institutional comparisons to achieve a more holistic understanding of the implications of physician oversupply.

## Supplementary material

Supplementary Figure S1 Geographic distribution of medical doctors per 10,000 inhabitants in Ecuador from 2006–2020. The maps illustrate the progressive increase in physician density across the country, with darker shades indicating higher physician rates.
