# Supplementary figures and images for: The growing oversupply of physicians in Ecuador: challenges and implications for the healthcare system
Source: Front Public Health. 2025 Aug 18;13:1605845. doi: 10.3389/fpubh.2025.1605845 (PMC12399641; doi:10.3389/fpubh.2025.1605845)

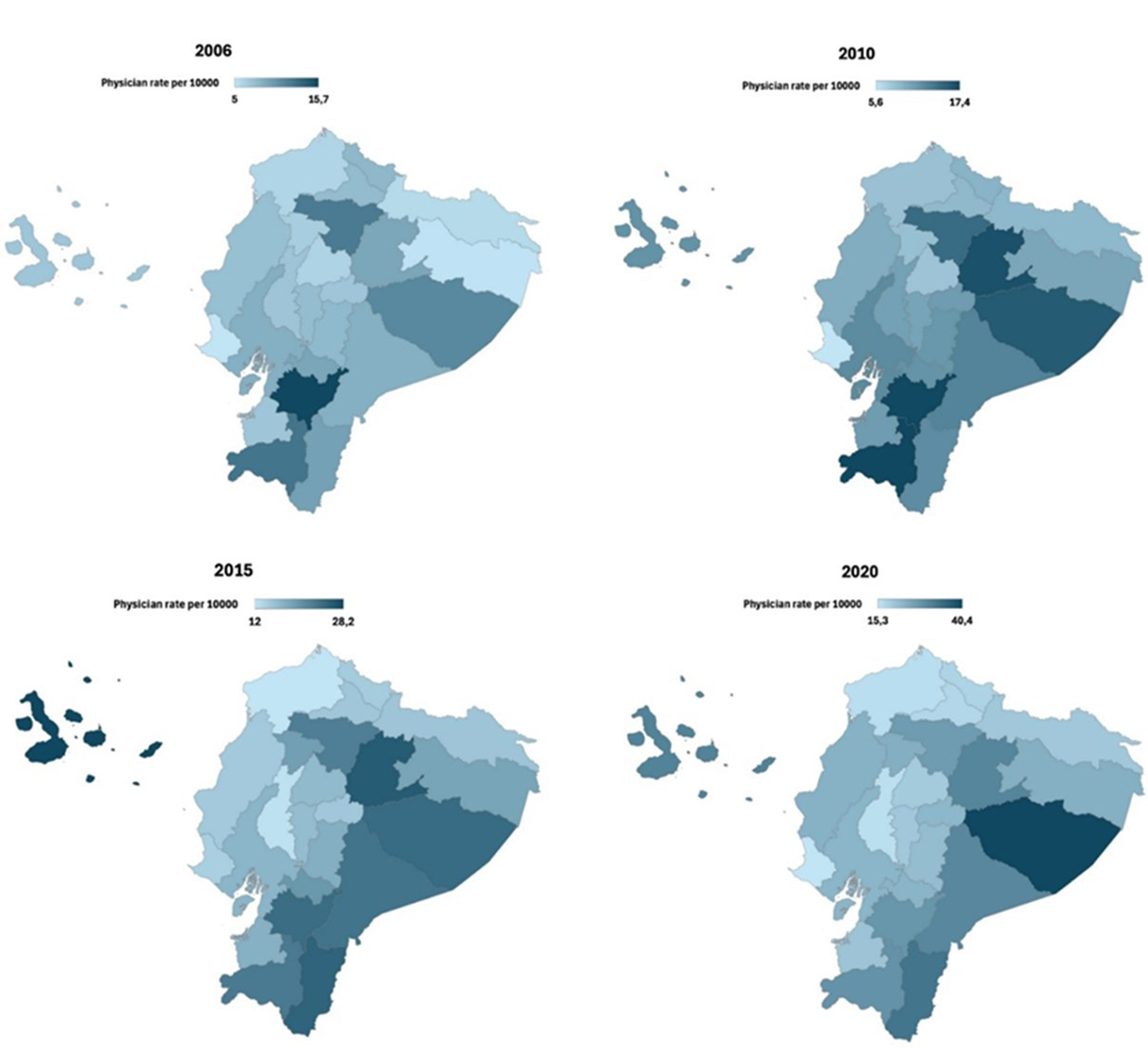

Supplement: Supplementary Figure S1 — Geographic distribution of medical doctors per 10,000 inhabitants in Ecuador from 2006–2020. The maps illustrate the progressive increase in physician density across the country, with darker shades indicating higher physician rates. [file Image_1.jpeg]
